# Serial C-reactive Protein as a Sensitive Marker of Treatment Response and Disease Severity in Acute Hand Infections

**DOI:** 10.7759/cureus.99594

**Published:** 2025-12-19

**Authors:** Sunandan Datta, Bratati Bandyopadhyay, Lydia S Abraham, Rahul H Shah, Mustafa Al-Jaafar, Pelumi Tawose, Manula Wijewardene

**Affiliations:** 1 Trauma and Orthopaedics, Aneurin Bevan University Health Board, Newport, GBR; 2 Trauma and Orthopaedics, Royal Gwent Hospital, Newport, GBR; 3 Trauma and Orthopaedics, The Grange University Hospital, Cwmbran, GBR

**Keywords:** c-reactive protein (crp), flexor sheath infections, hand infection, infection control measures, orthopedic hand surgery, paronychia, upper limb infections, wbc

## Abstract

Introduction: Acute hand infections are a frequent cause of emergency surgical admission and can lead to significant morbidity. C‑reactive protein (CRP) and white blood cell (WBC) count are commonly used biomarkers, yet their comparative value for monitoring treatment response remains unclear.

Methods: We conducted a retrospective cohort study of 69 consecutive patients with culture‑positive acute hand infections admitted between January and May 2024 at a single tertiary centre. Demographics, comorbidities, infection type, microbiology, management strategy, and outcomes were collected. CRP and WBC were recorded at admission and serially throughout treatment. Trends were analysed in relation to infection type and clinical response.

Results: The cohort was predominantly male, with 51 patients (73.9%), and diabetes mellitus was the most common comorbidity, being present in 22 patients (31.9%). *Staphylococcus aureus* was the leading pathogen. Elevated CRP (>10 mg/L) was present in 62 patients (89.86%) on admission, while leukocytosis was observed in 16 patients (23.2%). Mean CRP declined significantly from 72.4 mg/L at admission to 18.6 mg/L by discharge (p<0.001), whereas WBC changes were not statistically significant (p=0.184). Deep infections demonstrated significantly higher admission CRP levels than superficial infections (112.3 mg/L vs. 38.7 mg/L; p=0.002).

Conclusions: Serial CRP measurement is a more sensitive marker of treatment response in acute hand infections than WBC. Higher admission CRP correlates with deeper infections and longer hospital stay. Incorporating serial CRP monitoring into routine care may support antibiotic stewardship, discharge planning, and resource utilisation.

## Introduction

Acute hand infections are a frequent cause of emergency presentation and surgical admission and carry a risk of significant functional impairment if inadequately treated. Owing to the complex anatomy of the hand, with its confined fascial compartments, tendon sheaths, and close proximity to neurovascular structures, delayed or inadequate management can lead to rapid progression, tissue destruction, and long-term functional impairment. About two-thirds of these infections occur in men, with an average age of 40 years. Common causes include traumatic inoculation, domestic animal bites, occupational injuries, and, increasingly, injuries associated with do‑it‑yourself (DIY) activities observed since the COVID‑19 pandemic. Globally, *Staphylococcus aureus* remains the predominant organism, followed by *Streptococcus* species, with methicillin-resistant *S. aureus* (MRSA) posing an emerging challenge in urban regions [1-5]. *S. aureus *is the most commonly isolated microbe in acute hand infections, followed by *Streptococcus* species. MRSA is increasingly reported in urban regions and has been documented to account for up to 50% of cases in some studies [6,7].

Anatomically, hand infections can be classified into superficial and deep infections. While superficial infections are confined to the cutaneous and subcutaneous layers, deep infections extend to underlying structures, potentially compromising tendon sheaths, adjacent compartments, deep fascial planes, bursae, articular spaces, and bone [8].

Prompt recognition of the clinical signs and symptoms followed by early intervention is therefore critical. Clinical evaluation requires a detailed history of trauma and comorbidities, alongside an examination for erythema, oedema, and restricted motion. Identifying specific findings, such as Kanavel’s cardinal signs for flexor tenosynovitis, is vital for diagnosis. However, objective biomarkers like erythrocyte sedimentation rate (ESR), C-reactive protein (CRP), and white blood cell (WBC) counts routinely support decision-making. While elevated WBC and CRP at presentation may suggest infection, their role in monitoring response to therapy and informing specific treatment decisions (e.g., timing of discharge, switch from intravenous to oral antibiotics, or decision for re-operation) is less well defined in hand infections [9-13].

Existing literature on musculoskeletal and soft tissue infections suggests that CRP may be more sensitive than WBC and ESR for detecting ongoing inflammation and tracking treatment response, but data specifically focused on acute hand infections are limited [11,12].

This study aims to evaluate the clinical usefulness of serial CRP and WBC monitoring in culture‑positive acute hand infections and to determine whether biomarker profiles correlate with infection depth and treatment response.

## Materials and methods

Study design and setting

This single‑centre retrospective cohort study examined patients admitted with acute hand infections to the Department of Trauma and Orthopaedics at Grange University Hospital between January 1 and May 31, 2024. The study received approval as a service‑improvement audit via the Audit Management and Tracking (AMAT) platform (Audit Code: T&O/2025-26/19).

Patient selection and data collection

Electronic medical records on the Clinical Work Station (CWS) were queried to identify all patients presenting to the Emergency Department or Surgical Assessment Unit of our hospital, having the following inclusion and exclusion criteria.

Inclusion Criteria

Inclusion criteria included patients with a clinical diagnosis of acute hand infection involving the soft tissues of the hand and/or wrist, with culture-positive microbiological confirmation from wound, pus, tissue, or synovial fluid samples.

Exclusion Criteria

Exclusion criteria included the absence of positive microbiological cultures; chronic or recurrent infections (defined as symptom duration longer than four weeks or prior infection at the same site within three months); immunocompromised conditions likely to significantly distort biomarker responses (such as active chemotherapy, advanced HIV infection, or long-term systemic corticosteroid use beyond standard diabetic management); and incomplete biomarker data, including missing baseline or serial CRP or WBC measurements.

A total of 69 patients met the inclusion criteria. For each of these patients, demographic data, serial WBC and CRP counts, as well as comorbidities, microbiology reports, nature and site of infection and type of management were documented on a Microsoft Excel spreadsheet (Microsoft Corp., Redmond, WA, USA) for ease of analysis.

Elevated CRP was defined as >10 mg/L, and elevated WBC was defined as >11 × 10^9^/L, consistent with institutional reference thresholds.

Statistical analysis

Continuous variables (e.g., CRP, WBC, age) were reported as means and analysed using the Mann-Whitney U test for comparisons between groups (deep vs. superficial) and the Wilcoxon signed-rank test for paired data (admission vs. discharge trends). Categorical variables (e.g., gender, comorbidities) were assessed using Fisher's exact test. Statistical significance was defined as p<0.05. All analyses were performed using IBM SPSS Statistics for Windows, Version 28 (Released 2021; IBM Corp., Armonk, New York, United States).

## Results

Patient characteristics

**Figure 1:**
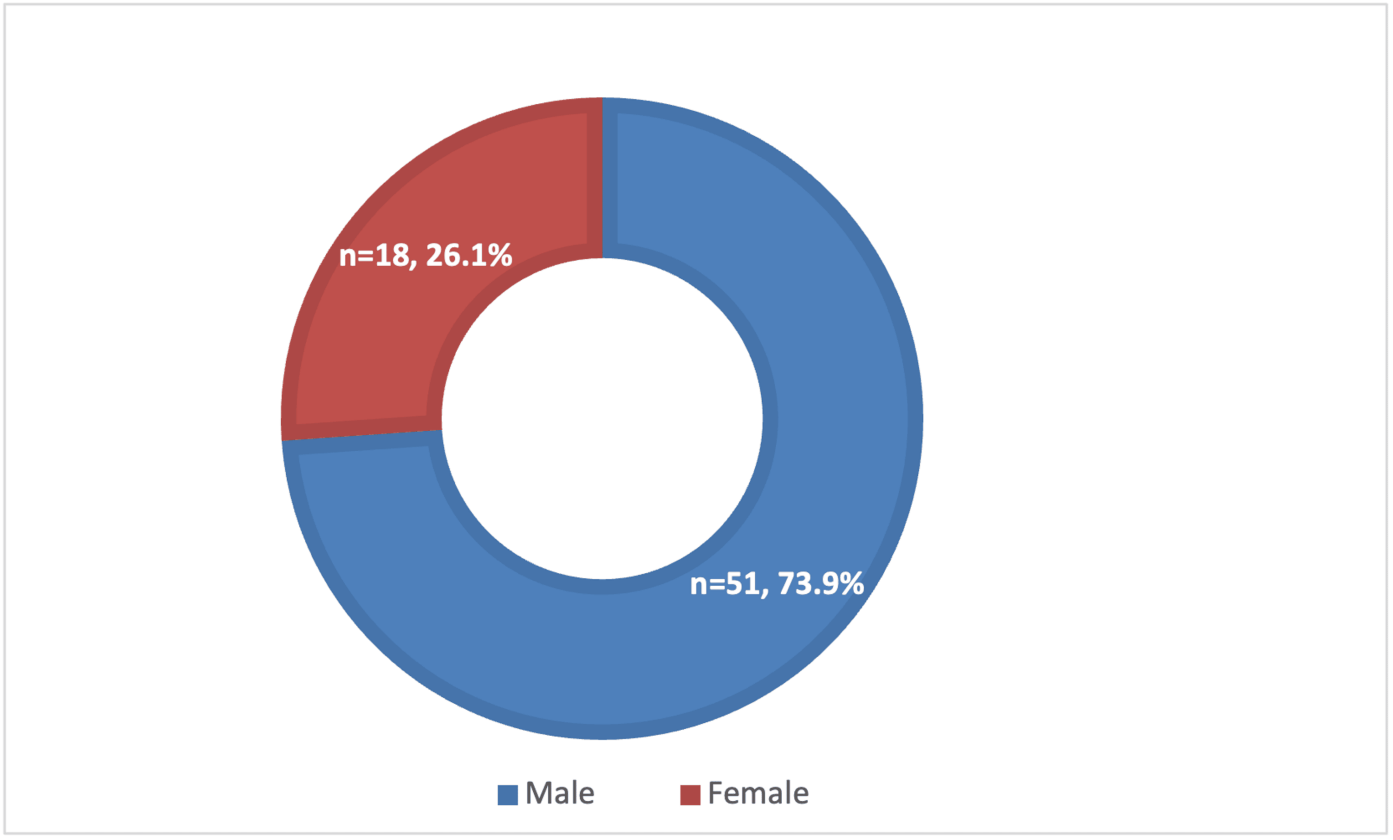
Gender Distribution of Patients With Acute Hand Infections

A total of 69 patients with culture-positive acute hand infections were included in the study. The cohort was predominantly male (n=51; 73.9%), with a mean age of 54.2 years (range: 17.6-89.7 years). Diabetes mellitus was the most prevalent comorbidity, present in 22 patients (31.9%). Additional comorbidities included hypertension, smoking, and peripheral vascular disease. The gender distribution is illustrated in Figure 1.

Infection mechanisms

**Figure 2:**
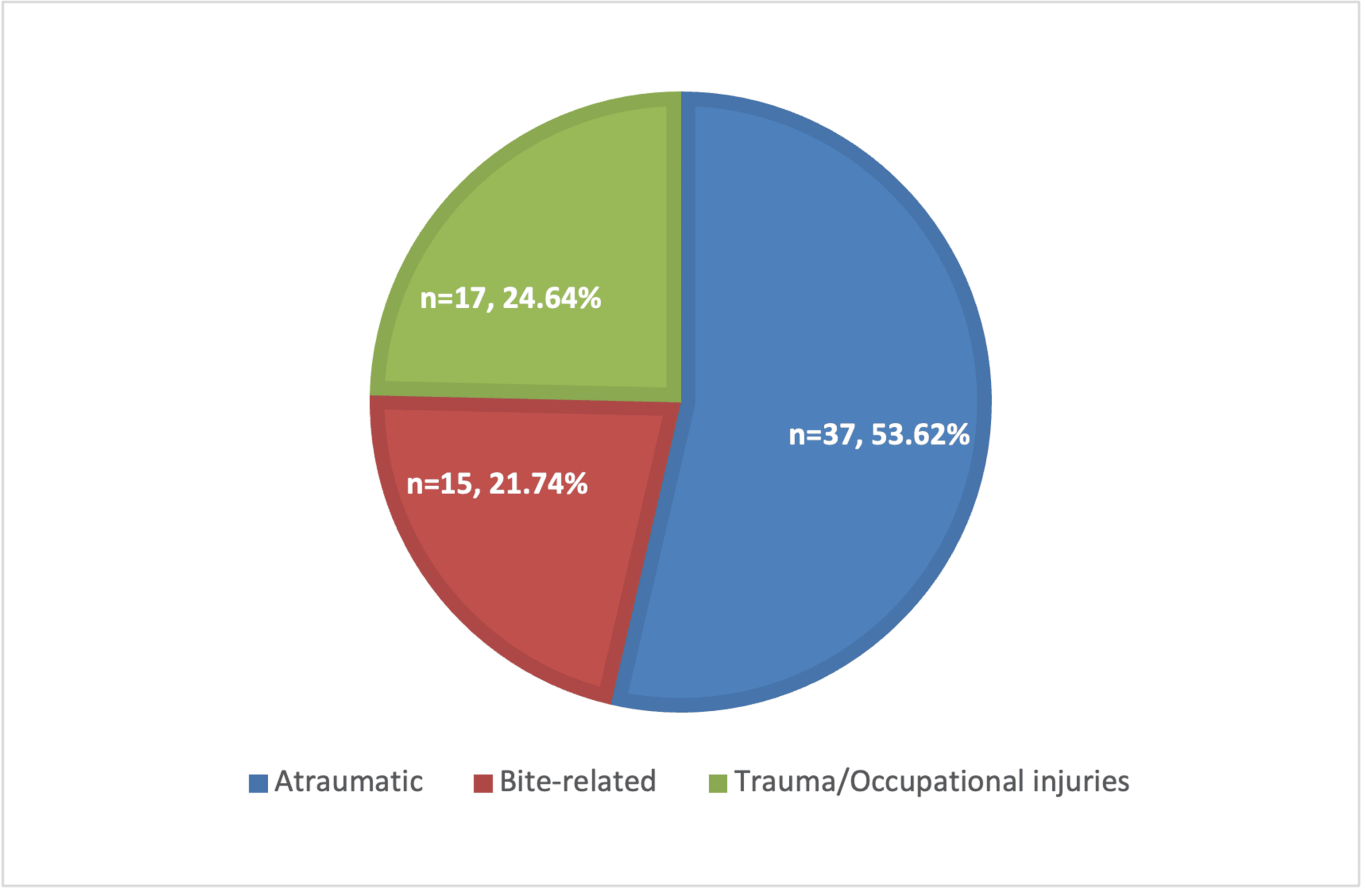
Distribution of Hand Infections by Etiological Mechanism The pie chart illustrates the classification of hand infection mechanisms in the study cohort (n=69). The majority of infections were classified as atraumatic, followed by trauma/occupational injuries and bite-related infections.

Mechanisms of infection included definite injury events (e.g., animal bites, traumatic inoculation, or occupational injuries) and non-traumatic etiologies (e.g., paronychia and flexor sheath infections). The most commonly involved anatomical regions were the digits, dorsum, palm, and thumb-index web space. Detailed infection mechanisms are presented in Table 1 and Figure 2.

**Table 1:** Mechanism of Hand Infections Among Study Patients (n=69) This table summarises the distribution of acute hand infections according to their mechanism of injury. Values are presented as the number of cases (n) and the percentage of the total cohort (%).

Category	Number, n	Percentage, %
Atraumatic	37	53.62
Bite-related	15	21.74
Trauma/occupational injuries	17	24.64

Microbiology

*S. aureus* was the predominant pathogen, isolated in 52 of the 69 culture-positive cases (75.4%), followed by *Streptococcus pyogenes* in 21 cases (30.4%). Other isolated organisms included *Streptococcus dysgalactiae*, Group B *Streptococcus*, *Pasteurella multocida, Proteus mirabilis, Clostridium perfringens*, and mixed anaerobes. Monomicrobial infections accounted for 38 cases (55.1%), while 31 cases (44.92%) were polymicrobial.

Management

Surgical intervention was required in 28 patients (40.58%), while 41 patients (59.42%) were managed with medical therapy alone. Surgical procedures included incision and drainage of abscesses, debridement of necrotic tissue, flexor sheath washout for pyogenic flexor tenosynovitis, and joint washout for septic arthritis. Antibiotic regimens were tailored according to culture results and sensitivities, following initial empirical coverage based on institutional guidelines.

Infection type and serial biomarker trends

Among the 69 patients, 25 (36.2%) were classified as having deep-seated infections, while 44 (63.8%) had superficial infections. At admission, CRP was elevated (>10 mg/L) in 62 patients (89.86%), with a mean admission value of 72.4 mg/L. Leukocytosis (WBC>11×10^9^/L) was observed in 16 patients (23.2%) on admission.

Deep-seated infections were associated with higher CRP on admission (112.3 mg/L vs. 38.7 mg/L in superficial infections; p=0.002), a more frequent requirement for surgical intervention, and a longer hospital stay (mean: 3.16 days) compared with 2.12 days for superficial infections. While admission WBC values trended higher in deep infections, this difference did not consistently achieve statistical significance (p=0.076).

Mean CRP decreased from 72.4 mg/L at admission to 18.6 mg/L on the date of discharge, which demonstrated a statistically significant decline (p<0.001). In contrast, WBC values displayed greater variability and less distinct trends. Reductions in mean WBC over the same interval did not reach statistical significance (p=0.184).

Patients with persistent or rising CRP levels beyond 48-72 hours were more likely to experience an adverse clinical course, although the relatively small sample size limited formal subgroup analysis.

## Discussion

This study demonstrates that serial CRP monitoring is more sensitive than the WBC count for tracking clinical improvement in acute hand infections. CRP’s rapid synthesis in response to inflammatory signalling and its relatively short half-life make it a more responsive biomarker than the WBC count, which may remain normal or fluctuate due to physiological influences unrelated to infection control.

The finding that CRP levels fall significantly (p<0.001) by discharge in clinical responders, whereas WBC counts do not show a statistically significant reduction (p=0.184), underscores the limitations of relying solely on leukocytosis as a marker of resolution. This aligns with broader orthopaedic literature suggesting that WBC counts can remain normal or equivocal even in the presence of significant localised abscesses or septic arthritis. The kinetic profile of CRP, with its rapid synthesis by the liver in response to interleukin-6 and its short half-life (approximately 19 hours), establishes it as a more responsive gauge of acute inflammation than cellular counts, which may be influenced by demargination or hydration status rather than effective infection control alone [11,12].

A study by Gauger et al. supports the efficacy of CRP as a biomarker for both the diagnosis and treatment of hand infections, concluding that CRP is more sensitive and responds more rapidly than ESR for monitoring the course of infection and response to treatment. However, this finding contradicts a paper by Houshian et al., who concluded that ESR was a better marker for monitoring hand infections, as it was elevated in 50% of symptomatic patients versus only 25% with raised CRP. Because the mean duration of follow-up in their study was significantly longer than ours, the discrepancy is possibly due to ESR remaining elevated longer than CRP due to its longer half-life [13-16].

Our cohort confirmed* S. aureus* as the predominant pathogen, consistent with global epidemiological data on hand infections, followed by *S. pyogenes* [7,14].

Notably, patients with deep-space infections (such as flexor tenosynovitis or deep palmar space abscesses) presented with significantly higher admission CRP levels (112.3 mg/L) compared to those with superficial infections like acute paronychia and felon. In our study, patients with superficial hand infections responded well to oral antibiotics, which is reassuring. This suggests that admission CRP could serve as a valuable adjunct to physical examination, specifically Kanavel’s signs or assessment of sheath tenderness in stratifying risk. A markedly elevated CRP at presentation should heighten the suspicion for deep structural involvement, potentially prompting earlier surgical decompression rather than a trial of antibiotics alone. These findings are echoed by several studies on a similar topic, which highlight the role of CRP as a useful adjunct in both diagnosis and monitoring of disease progression [17-20].

The clinical utility of these findings lies in discharge planning and antibiotic stewardship. In an era of increasing antimicrobial resistance, the decision to switch from intravenous to oral antibiotics or to discharge a patient often relies on a combination of clinical appearance and biochemical trends. Our study suggests that a down-trending CRP is a reassuring marker of source control, whereas a static WBC count should not necessarily delay discharge if the patient is clinically improving. Conversely, a failure of CRP to decrease by day 3 post-intervention may serve as an early warning for ongoing infection and the need for repeat surgical debridement. In this regard, we recommend using CRP in conjunction with the WBC count as a biomarker for monitoring infection and treatment response [21,22].

Despite our best efforts, several limitations need to be acknowledged. This was a single-centre retrospective review with a modest sample size, which limits the power to perform detailed subgroup analyses between specific bacteria types. Additionally, by including only culture-positive cases, we may have excluded milder cellulitis cases that resolved with antibiotics before a sample could be obtained, potentially skewing our baseline biomarkers toward more severe infections. We did not routinely order ESR values for all of our patients, which is why they were not included in this comparative analysis of biomarkers.

## Conclusions

In conclusion, while clinical assessment remains the cornerstone of diagnosis, serial CRP monitoring is a more valuable tool than WBC for tracking the resolution of acute hand infections. Incorporating routine CRP trends can effectively support antibiotic stewardship, aid in distinguishing infection depth and facilitate safe, timely discharge planning in hand surgery patients. Future prospective studies should establish precise CRP cut-off values to formalise algorithms for the switch to oral antibiotics in hand infection patients.
